# Ethyl Acetate Extract of Marine Algae, *Halymenia durvillei,* Provides Photoprotection against UV-Exposure in L929 and HaCaT Cells

**DOI:** 10.3390/md20110707

**Published:** 2022-11-11

**Authors:** Pichnaree Kraokaew, Preeyanuch Manohong, Prachayaporn Prasertsuksri, Prapaporn Jattujan, Nakhon Niamnont, Montakan Tamtin, Prasert Sobhon, Krai Meemon

**Affiliations:** 1 Department of Anatomy, Faculty of Science, Mahidol University, Ratchathewi, Bangkok 10400, Thailand; 2 Department of Chemistry, Faculty of Science, King Mongkut’s University of Technology Thonburi, Bang Mod, Bangkok 10140, Thailand; 3 Department of Anatomy, Faculty of Medicine, Khon Kaen University, Khon Kaen 40002, Thailand; 4 Kung Krabean Bay Royal Development Center, Department of Fisheries, Khlong Khut Sub-District, Tha Mai, Chantaburi 22000, Thailand

**Keywords:** *Halymenia durvillei*, photoaging, ultraviolet A (UVA), ultraviolet B (UVB), antioxidant, NF-E2-related factor 2 (Nrf2), matrix metalloproteinases (MMPs), procollagen

## Abstract

*Halymenia durvillei* is a red alga distributed along the coasts of Southeast Asian countries including Thailand. Previous studies have shown that an ethyl acetate fraction of *H. durvillei* (HDEA), containing major compounds including n-hexadecanoic acid, 2-butyl-5-hexyloctahydro-1H-indene, 3-(hydroxyacetyl) indole and indole-3-carboxylic acid, possesses high antioxidant and anti-lung cancer activities. The present study demonstrated that HDEA could protect mouse skin fibroblasts (L929) and human immortalized keratinocytes (HaCaT) against photoaging due to ultraviolet A and B (UVA and UVB) by reducing intracellular reactive oxygen species (ROS) and expressions of matrix metalloproteinases (MMP1 and MMP3), as well as increasing Nrf2 nuclear translocation, upregulations of mRNA transcripts of antioxidant enzymes, including superoxide dismutase (*SOD*), heme oxygenase (*HMOX*) and glutathione S-transferase pi1 (*GSTP1*), and procollagen synthesis. The results indicate that HDEA has the potential to protect skin cells from UV irradiation through the activation of the Nrf2 pathway, which leads to decreasing intracellular ROS and MMP production, along with the restoration of skin collagen.

## 1. Introduction

Solar UV radiation reaching the earth is a continuous spectrum of electromagnetic radiation ranging from 280 to 400 nm (UVA (320 to 400 nm) and UVB (280 to 320 nm)). UVA radiation exposed to the skin mostly reaches the deep dermis (20–30%) and is partially absorbed by the epidermis. UVB radiation is mostly absorbed by the epidermis and only 10% penetrates into the dermis. Therefore, UVA has major action on the dermis rather than UVB which also causes skin photoaging. In addition, exposure to UVB markedly resulted in erythema (sunburn), pigmentation (tanning), edema, skinfold thickening, sunburn cells (SBC) formation, phototoxic reactions, photosensitivity, photocarcinogenesis and photoimmunosuppression [1,2].

Photoaging is a serious health issue worldwide; its undesirable consequences are sunburn, skin wrinkles, rapid skin turnover, and lower regenerative potential which leads to abnormal skin physiology and pathology, especially skin aging and cancer [3]. Photoaging is caused by accumulative effects of UV exposure which generate free radicals, especially reactive oxygen species (ROS) that induce photoaging processes. ROS stimulate the upregulation and release of matrix metalloproteinases (MMPs) from fibroblasts and keratinocytes to degrade extracellular matrix (ECM) components, such as collagen and elastin, leading to impairments in skin structure and its barrier function. UV also reduces collagen synthesis, resulting in an imbalance between collagen production and degradation [4].

To prevent skin photoaging, several methods have been applied, for example, using sunscreens containing UV-filtering compounds and certain medications, such as retinoid and its derivatives including tretinoin and tazarotene [5]. However, some adverse effects of topical retinoids including skin irritation, peeling, erythema and dryness, have been reported [6]. In addition, retinoid is an inhibitor of NF-E2-related factor 2 (Nrf2) and it markedly reduced the binding between Nrf2 to ARE enhancer, which consequently reduces the expressions of antioxidant genes [7].

Nrf2 is a key transcription factor that upregulates the expressions of antioxidant enzymes including superoxide dismutase (SOD1), catalase (CAT), glutathione S-transferase (GST), and heme oxygenase (HO-1) which neutralize ROS [8,9,10]. Moreover, Nrf2 could attenuate UV-induced ROS generation and damage by maintaining high levels of antioxidants in the skin. It has been shown that UVB-irradiated Nrf2-knockout mice exhibited more apoptotic cells, accumulation of oxidized DNA, breakdown of extracellular matrix, erythema and coarse wrinkle formation, compared to wild-type mice subjected to similar treatment [11,12]. Moreover, silencing Nrf2 significantly increased UVA-induced cell damage in skin keratinocytes, while activation of Nrf2 attenuated UVA-mediated MMP-1 upregulation [13,14]. Therefore, targeting Nrf2 with phytochemical compounds has been proposed as a pharmacological strategy for preventing skin photoaging.

Marine microalgae and macroalgae possess a variety of biologically active compounds, many of which are unique and different from those of land plants. Because of extreme environments, including high oxygen concentration, salinity and sunlight with strong UV, algae modify their metabolisms to protect themselves from the elements by generating various antioxidants in the forms of secondary metabolites [15,16]. Accordingly, algae-derived compounds have gained attention in the last few years as antioxidant sources, Nrf2 inducers, and photo-protective compounds against UV exposure [17,18,19,20,21,22,23].

*Halymenia durvillei* (HD) is belonged to Rhodophyta, the division of red algae, having the large and bushy thalli with dark red color and soft cartilaginous texture. HD is locally distributed in Western Pacific and the Indomalayan Archipelago, including Thailand, Vietnam, and the Philippines [24]. There are few biological activities of HD that have been reported nowadays. The polysaccharide-rich extract of HD could attenuate red skin conditions and able to modulate the skin microbiota associated with aging skin [25]. The acidified methanol fraction of HD exhibited alpha-amylase, elastase, and tyrosinase inhibitions [26]. Additionally, it was found that the ethyl acetate extract of HD contains high antioxidant activity [19]. However, the protective effect of HDEA against UV-induced skin photoaging has not yet been investigated. In the present study, we demonstrated the positive potential of HDEA in protecting skin cells, fibroblast (L929) and keratinocyte (HaCaT), against UV-induced photoaging.

## 2. Results

### 2.1. Gas Chromatography-Mass Spectrometry (GC-MS) of HDEA

**Figure 1:**
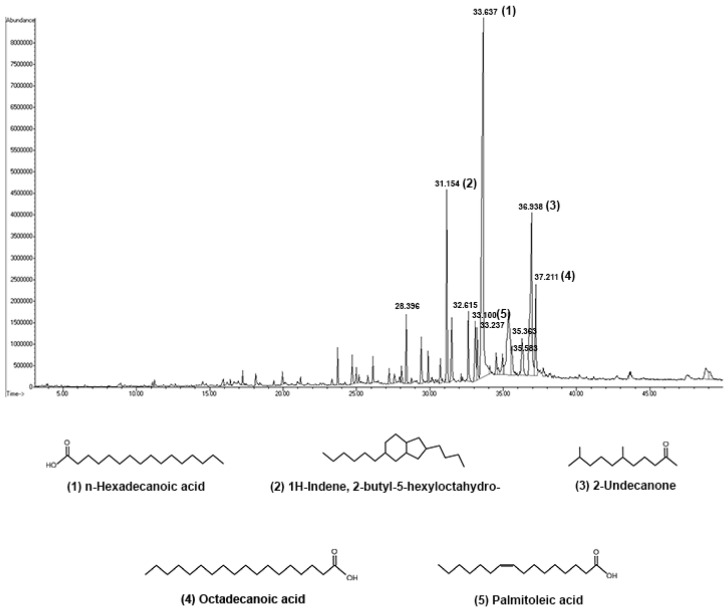
GC-MS Chromatogram of HDEA; five major compounds include (1) n-Hexadecanoic acid, (2) 2-Butyl-5-hexyloctahydro-1H-indene, (3) 6,10-Dimethyl-2-undecanone, (4) Octadecanoic acid and (5) Palmitoleic acid.

GC-MS was performed to identify compounds presented in HDEA (Figure 1). A total of 24 compounds were identified in HDEA and have been characterized in our previous study [19]. The major constituents that contain a relative peak area greater than 2% were highlighted in Table 1. GC-MS analysis showed that the major components in HDEA were n-Hexadecanoic acid (34.09%), 2-Butyl-5-hexyloctahydro-1H-indene (16.63%), 6,10-Dimethyl-2-undecanone (7.54%), Octadecanoic acid and (4.6%) and Palmitoleic acid (3.75%).

### 2.2. HDEA Increases Cell Viability in UV-Induced L929 and HaCaT Cells

**Figure 2:**
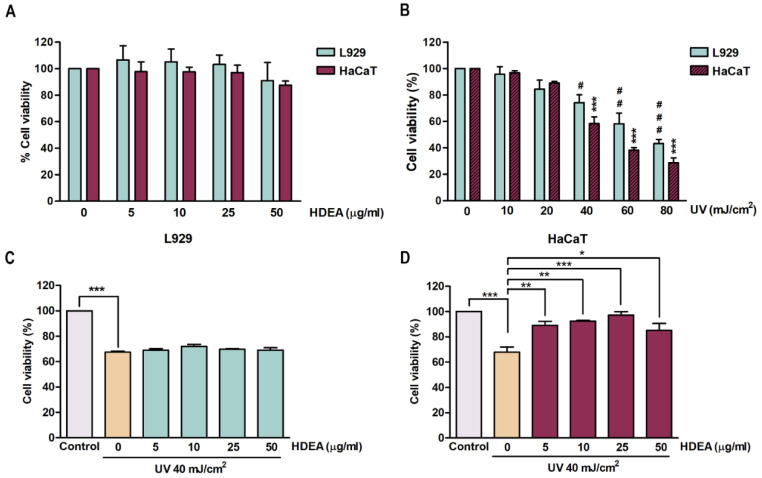
Effects of HDEA treatment on cell cytotoxicity and viability of L929 (blue bars) and HaCaT (purple bars) cells at 24 h with and without UV irradiation as determined by MTT assay. (**A**) Effects of HDEA (0, 5, 10, 25, 50 μg/mL) on cell viability of L929 and HaCaT cells. (**B**) Effects of serial doses (0, 10, 20, 40, 60 and 80 mJ/cm^2^) of UV on viability of L929 and HaCaT cells compared to control group (no UV irradiation) with levels of significant difference as indicated by # *p* < 0.05, ## *p* < 0.01 and ### *p* < 0.001 for HaCaT and *** *p* < 0.001 for L929. (**C**) The protective effect of HDEA on UV-irradiated L929 cells. (**D**) The protective effect of HDEA on UV-irradiated HaCaT cells. The numbers of asterisk denoted the levels of significant difference (* *p* < 0.05, ** *p* <0.01, *** *p* < 0.001).

Cells were treated with HDEA at different doses (5, 10, 25 and 50 μg/mL) for 24 h and evaluated for cell viability using an MTT assay. Figure 2A showed that HDEA was not cytotoxic at all the doses tested in both L929 and HaCaT cells. Therefore, we used all four doses of HDEA for further studies. In order to determine the appropriate dose of UV, we first exposed the cells to different doses of UV (0, 10, 20, 40, 60 and 80 mJ/cm^2^). As shown in Figure 2B, cell viability of both L929 and HaCaT cells was reduced in a dose-dependent manner (EC_50_ (half maximal effective concentration) = 71.33 and 48.89 mJ/cm^2^, respectively); thus, for subsequent experiments, we chose UV intensity at 40 mJ/cm^2^ which was the dose proven to affect the cells but still maintained 60% viable cells. Figure 2C,D showed that UV exposure reduced the viability in both L929 and HaCaT cells up to 67.49 ± 0.74% and 67.89 ± 3.38%, respectively, compared to untreated and unexposed cells. When the cells were treated with HDEA for 24 h and then irradiated with UV the viability of HaCaT cells at all doses of HDEA significantly increased compared to untreated and UV-exposed cells. However, L929 cells treated with HDEA and irradiated with UV exhibited a slightly increased cell viability which was not insignificant different from the control.

### 2.3. HDEA Attenuate Intracellular ROS Level in UV-Induced Cells

**Figure 3:**
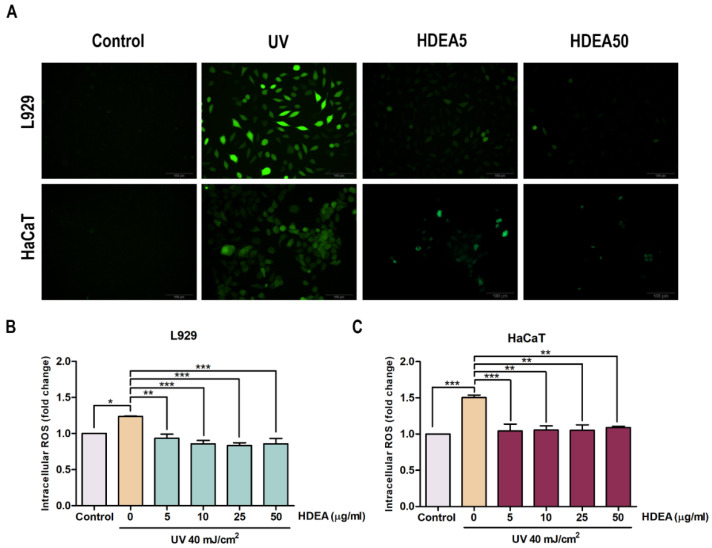
Effect of HDEA on intracellular ROS generation induced by UV in L929 and HaCaT cells. (**A**) Cells were treated with HDEA for 24 h before being exposed to UV. Then DCF fluorescence was measured after 45 min of DCFH-DA incubation by using IX83 Olympus inverted fluorescence microscope. (**B**) The relative DCF fluorescence intensity of L929 and (**C**) the relative DCF fluorescence intensity of HaCaT. The numbers of asterisk denoted the levels of significant difference (* *p* < 0.05, ** *p* <0.01, *** *p* < 0.001).

The intracellular ROS levels of UV-exposed cells were determined by Live Cell imaging (Figure 3A) and detected by a microplate reader. ROS levels were increased up to 1.24 and 1.5 fold compared to controls in L929 (Figure 3B) and HaCaT (Figure 3C) cells, respectively. Pre-treatment of HDEA significantly reduced ROS levels caused by UV exposure in all doses in both L929 and HaCaT cells. Therefore, we decided to use only the lowest and highest doses, which are 5 and 50 μg/mL, in further experiments. The results demonstrated that HDEA has a ROS scavenging effect following UV exposure in both L929 and HaCaT cells.

### 2.4. HDEA Promotes Nrf2 Nuclear Translocation

**Figure 4:**
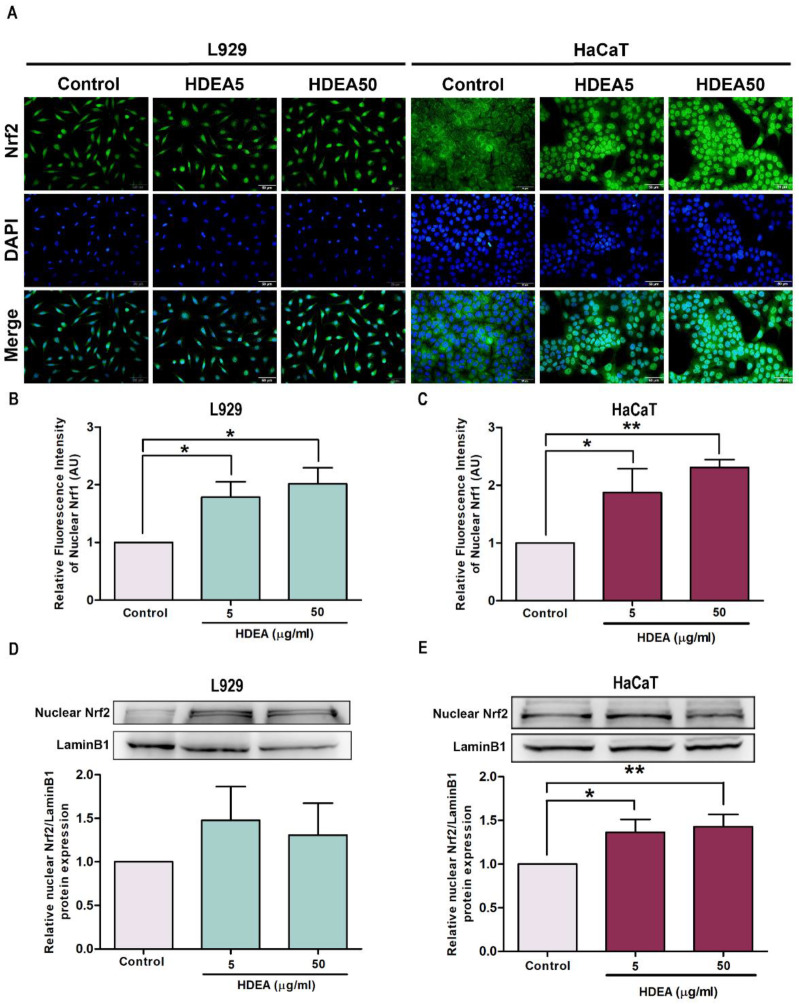
Effect of HDEA treatments (at 5 and 50 μg/mL) on Nrf2 nuclear translocation. (**A**) L929 and HaCaT cells were treated with HDEA, then the localization of Nrf2 was observed by fluorescence microscope (scale bar = 50 μm). Quantification of fluorescence indicative of nuclear Nrf2 localization by Image J in L929 cell (**B**) and HaCaT (**C**). Western blot analyses of Nrf2 nuclear localization in L929 cell (**D**) and HaCaT cell (**E**) treated with HDEA. Different numbers of asterisks denoted the levels of significant difference (* *p* < 0.05 and ** *p* <0.01).

To examine whether HDEA activates Nrf2 nuclear translocation that leads to the activation of antioxidant genes, we investigated such translocation using immunofluorescence and Western blot analysis. The fluorescence intensity appearing in L929 and HaCaT cells treated with HDEA showed increased levels of Nrf2 inside the nucleus (Figure 4A). As shown in Figure 4B,C, the intensities of Nrf2 accumulation in the nucleus of L929 and HaCaT treated with HDEA at doses of 50 μg/mL were significantly increased up to 2.02 and 2.55 fold, respectively, compared to the untreated control groups. Similarly, in Western blot analysis, HDEA-treated HaCaT cells showed significantly enhanced levels of nuclear Nrf2 up to 1.36 and 1.42 fold when treated with doses 5 and 50 μg/mL, respectively, compared to the untreated control (Figure 4E). However, L929 showed a slight but not significant increase in Nrf2 nuclear accumulation (Figure 4D). Therefore, the results indicated that HDEA enhanced Nrf2 nuclear translocation in all tested cells, especially in HaCaT.

### 2.5. HDEA Upregulates mRNA Levels of Nrf2-Targeted Genes Encoding Antioxidant Enzymes in UV-Irradiated Skin Cells

**Figure 5:**
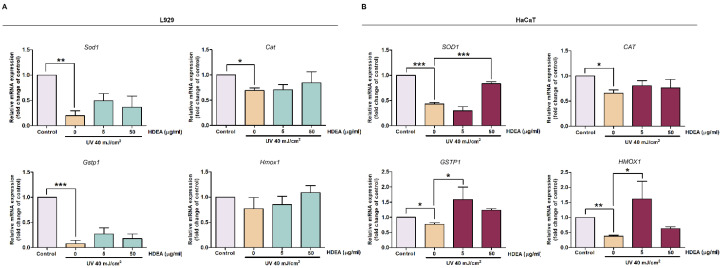
Effect of HDEA treatments on levels of mRNA encoding antioxidant enzymes in UV-exposed cells as quantified by RT-qPCR. (**A**) mRNA levels of *Sod1*, *Cat*, *Gstp1* and *Hmox1* in L929 cells treated with HDEA. (**B**) mRNA levels of *SOD1*, *CAT*, *GSTP1* and *HMOX1* in HaCaT cells treated with HDEA. Different numbers of asterisks denoted the levels of significant difference (* *p* < 0.05, ** *p* <0.01, *** *p* < 0.001).

To evaluate the expression levels of antioxidant genes targeted by Nrf2, we used RT-qPCR to measure levels of mRNAs encoding antioxidant enzymes, including superoxide dismutase 1 (*SOD1* and *Sod1*), catalase (*CAT* and *Cat*), glutathione S transferase pi 1 (*GSTP1* and *Gstp1*) and heme oxygenase 1 (*HMOX1* and *Hmox1*). UV exposure reduced mRNA levels of *Sod1*, *Cat*, *Gstp1* and *Hmox1* down to 0.20, 0.69, 0.08 and 0.77 fold compared to control in L929 cells (Figure 5A). Pre-treatment with 50 μg/mL HDEA insignificantly increased mRNA levels of *Sod1*, *Cat*, *Gstp1* and *Hmox1* up to 0.38, 0.84, 0.17 and 1.09 fold compared to control, respectively. As expected, the levels of mRNAs encoding all antioxidant enzymes (*SOD1*, *CAT*, *GSTP1* and *HMOX1)* decreased in UV-irradiated HaCaT cells to 0.43, 0.73, 0.77 and 0.38 fold compared to the control group (Figure 5B). Notably, in HaCaT cells pre-treatment with HDEA at 5 μg/mL before UV exposure could recover the levels of mRNAs encoding *CAT*, *GSTP1* and *HMOX1* up to 0.82, 1.59 and 1.61 folds of the control group, respectively, but not for *SOD1* mRNA. However, pre-treatment of 50 μg/mL HDEA could significantly upregulate *SOD1* mRNA level to 0.83 fold of the control. Moreover, pre-treatment with 50 μg/mL HDEA was still able to slightly increase *CAT* and *GSTP1* up to 0.83 and 1.24 folds of the control, respectively. These results indicated that HDEA has a protective effect against UV irradiation by inducing the expressions of superoxide dismutase, glutathione-s-transferase, and heme oxygenase, especially in HaCaT which are keratinocytes.

### 2.6. HDEA Attenuates UV-Induced MMP1 and MMP3 Expressions in L929 and HaCaT Cells

**Figure 6:**
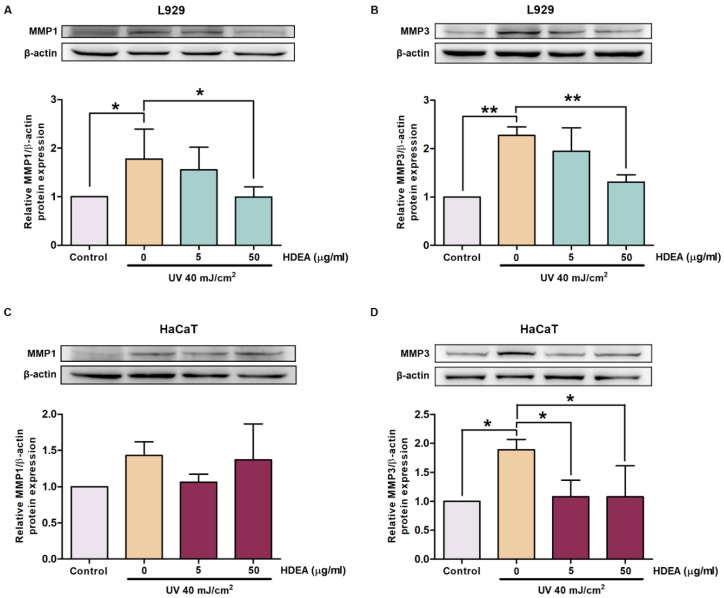
Protective effect of HDEA treatments against UV-induced MMPs production. Western blot analysis of MMP1 (**A**) and MMP3 (**B**) in L929 cell. Western blot analysis of MMP1 (**C**) and MMP3 (**D**) in HaCaT cell. Data are shown as mean ± SD (*n* = 3). Asterisks indicate significant differences compared with the control group (UV-irradiated group without HDEA treatment). * *p* < 0.05 and ** *p* < 0.01.

UV exposure induced expressions and accumulations of matrix metalloproteinases (MMPs) leading to increased ECM degradation and impaired skin barrier function, which finally resulted in wrinkle formation. We used Western blot analysis to measure the effects of HDEA on MMP protein expressions induced by UV irradiation. Figure 6A,B showed a significant increase in MMP1 and MMP3 expressions in UV-irradiated L929 cells up to 1.78 and 2.27 fold compared to that of the control group. Interestingly, pre-treatment with HDEA at a dose of 50 μg/mL could reduce MMP1 and MMP3 expression to 0.79 and 0.96 fold compared to the control. Similarly, UV-irradiated HaCaT cells showed an increase in MMP1 and MMP3 expressions up to 1.43 and 1.89 fold compared to the control (Figure 6C,D). Pre-treatment of HDEA at both 5 and 50 μg/mL decreased MMP3 to 1.08 fold compared to the control group. However, pre-treatment with 5 μg/mL HDEA slightly reduced MMP1 down to 1.06 fold compared to the control, which was not significantly different. These results demonstrated HDEA has a protective effect against UV-irradiation by reducing MMP1 and MMP3 expressions in both L929 and HaCaT cells, especially when treated with a dose of 50 μg/mL.

### 2.7. HDEA Improves Collagen Synthesis in UV-Irradiated L929 and HaCaT Cells

**Figure 7:**
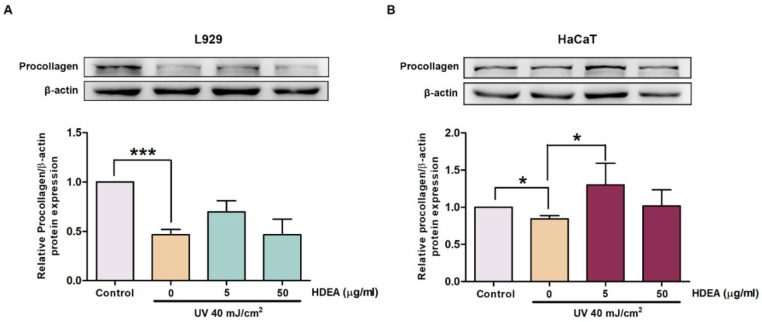
Effect of HDEA treatments on procollagen synthesis. (**A**) Western blot analysis of type I procollagen in L929 cell. (**B**) Western blot analysis of type I procollagen in HaCaT cell. Data are shown as mean ± SD (*n* = 3). Asterisks indicate levels of significant difference compared with control group (UV-irradiated group without HDEA treatment). * *p* < 0.05 and *** *p* < 0.001.

UV exposure reduced collagen synthesis, resulting in extreme loss of collagen fibers causing wrinkle formation. To elucidate whether HDEA treatments improved collagen synthesis, we measured procollagen type 1 by Western blot analysis. The results showed that procollagen synthesis in UV-irradiated L929 and HaCaT cells was dramatically decreased by 0.51 and 0.17 folds compared to the control (Figure 7A,B). Pre-treatment with HDEA at a dose of 5 μg/mL before exposure to UV could improve procollagen synthesis up to 0.91 and 1.19 fold of the control in L929 and HaCaT cells. These results indicated that pre-treatment with 5 μg/mL HDEA could restore procollagen levels in both L929 and HaCaT cells.

## 3. Discussion

Our present study is the first to substantiate the protective effect of *H. durvillei* against UVA- and UVB-induced photoaging in fibroblasts and keratinocytes. Here, we demonstrated that the ethyl acetate fraction of *H. durvillei* (HDEA) reduced UV-induced intracellular levels of ROS and the expression of MMPs. Moreover, HDEA treatment could also promote Nrf2 nuclear translocation, antioxidant gene expressions, and collagen production.

Ethyl acetate has been proven in cosmetic products since it has no negative effect on genotoxicity, skin irradiation, photoirritation and photoallergenicity [28]. Our previous study reported that HDEA contained higher total phenolic and flavonoid contents than butanol, hexane, ethanol and aqueous extracts, respectively. DPPH radical scavenging activity of HDEA also presented the greatest activity compared to other fractions. A lifespan assay in a *Caenorhabditis elegans* model also revealed that HDEA increased the longevity of *C. elegans* more than other fractions. GC-MS analysis of HDEA revealed several compounds, with the main components identified being n-hexadecanoic acid, 2-butyl-5-hexyloctahydro-1H-indene and octadecanoic acid [27]. A molecular docking study of hexadecanoic acid showed that it interacted with photoaging markers including activator protein1 (AP1), tumor necrosis factor-alpha (TNF-α) and Nuclear factor kappa B (NF-kB) through hydrogen bonding, which might suppress photoaging [29]. A Persian walnut, *Juglans regia* L., containing hexadecanoic acid as one of the major components also has a protective effect against UVB-induced photoaging by suppressing MMP expression and increasing procollagen production [30,31]. In addition, purple yam, *Dioscorea alata*, containing high amounts of hexadecanoic acid and octadecanoic acid, could protect *C. elegans* against α-synuclein protein aggregation through increasing SKN1/Nrf2 nuclear translocation and activation of antioxidant gene expressions [32]. However, other compounds that cannot be identified by GC-MS may possibly exert a protective effect against UV-induced photoaging in the study. For example, mycosporine-like amino acids (MAAs) are expected to be contained in HDEA with their moderately polar properties which could multiply the effect of HDEA on anti-photoaging as well as inhibit collagenase and elastase activities [33,34,35,36]. Therefore, these compounds, also found in HDEA, might be the active chemicals that protect skin cells against UV irradiation.

ROS are generated from the absorption of UV photons by cellular chromophores and photosensitizers, which are in a highly excited state and cause ROS formation. The increased reactive oxygen species further damage cellular macromolecules and interact with a number of regulatory pathways that consequently lead to skin photoaging [37]. As expected, intracellular levels of ROS in L929 and HaCaT cells were significantly increased after UVB exposure for 2 h, as reported in other studies [38,39,40]. Treatment with HDEA could reduce and maintain the intracellular level of ROS in the UV-irradiated cells close to that of the unexposed cells. These results demonstrated that HDEA could scavenge ROS and attenuate UV-mediated ROS production. This was supported by our previous study which used a DPPH radical scavenging assay to demonstrate that HDEA was the most active fraction with antioxidant activity compared to ethanol, hexane and butanol fractions of *H. durvillei* [27].

Increased ROS activate Nrf2, a key factor for cytoprotection against oxidative stress. Nrf2 acts as a transcriptional activator of genes encoding antioxidant proteins and phase 2 detoxifying enzymes. Under normal conditions, Nrf2 is located in the cytoplasm by interacting with Kelch-like ECH associating protein 1 (Keap1) which is a substrate-specific adaptor of Cul3-based E3 ubiquitin ligase. Subsequently, the complex is transferred to be degraded by the ubiquitin-proteasome pathway. Interaction with ROS or other inducers causes configuration changes in Keap1 resulting in dissociation of the Keap1–Nrf2 complex which allows Nrf2 to be free and translocated into the nucleus where it activates the expression of antioxidant genes, including superoxide dismutase, catalase and heme oxygenase, as well as detoxifying genes including glutathione-s-transferase pi1 [41,42]. These enzymes are depleted in UVA and UVB exposed cells, which contributes to the rise in oxidative stress and damage in the skin [38,43,44,45,46]. Many algae-derived compounds have been demonstrated to activate Nrf2 pathways, for instance, bromo-4, 5-dihydroxybenzaldehyde from marine red algae (*Rhodomela confervoides*, *Polysiphonia morrowii*, and *Polysiphonia urceolata*), ethanol extract of *Sargassum serratifolium* brown algae, and keto-fatty acid-based compounds from *Ulva lactuca* green algae [8,9,10]. In the present study, we found that the level of Nrf2 in the nucleus was significantly increased in HDEA-treated L929 and HaCaT cells. This led to significantly increased levels of *SOD1*, *GSTP1* and *HMOX1* mRNAs in UV-exposed HaCaT cells, while the levels of these mRNAs also increased in L929 but with no significant difference from the control. In a previous study, the kinetic analysis of intracellular ROS formation after UVB irradiation found that UVB triggered two phases of ROS production. The first wave of ROS rapidly increased after UVB irradiation and decreased continuously to baseline in an hour. The second wave took place after 3 h and was maintained at high levels for 6 h before slightly decreasing to baseline levels. The late ROS increase is involved with increased mitochondrial permeability and cytochrome c release leading to cellular apoptosis [47]. From our results after 6 h post-irradiation, antioxidant enzyme levels including *SOD1*, *GSTP1* and *HMOX1* in HaCaT still remained in higher levels compared to UV-exposing groups, while antioxidant enzyme levels in L929 were not significantly different suggesting that L929 cells might not have enough antioxidant enzymes to deal with the second wave of ROS production. The results explained cell viability assay in which treatment with a low dose of HDEA could not protect the L929 cells against UV exposure while increasing the dose or post-irradiation treatment could better protect L929 against UV exposure. Altogether, these results suggested that HDEA enhanced Nrf2 activation which led to the increased syntheses of antioxidant enzymes in HaCaT cells at the level more than in L929 cells.

UV-induced ROS also caused an imbalance between collagen degradation and collagen synthesis due to increased expressions of matrix metalloproteinase (MMPs), resulting in extreme loss of collagen and elastin, and premature wrinkle [48]. MMPs are a family of protease enzymes involved in the degradation and remodeling of the extracellular matrix (ECM). MMP1 has been identified as collagenase I which has an ability to degrade collagens types I, II, III, VII, VIII, X and proteoglycans, while MMP3 belongs to the stromelysins subfamily that degrades laminin, fibronectin, elastin, proteoglycans, collagens types III, IV, V, VII, IX and X [49]. Natural products derived from algae possess strong antioxidant properties that could mitigate MMPs upregulation by UV exposure [40,50,51,52,53]. In the present study, we found that HDEA treatment could prevent MMP1 and MMP3 production induced by UV exposure in both L929 and HaCaT cells. Our results are compatible with the study showing that GSTP1 is a negative regulator of the MAPK pathway which mediates MMPs production, thus it protects cells against apoptosis [54]. In particular, an increase in GSTP1 in HaCaT cells might explain the higher viability of HaCaT over L929 cells. As well, HDEA could stimulate HaCaT cells to produce more procollagen type I than L929 cells and significantly restored procollagen type I synthesis. Taken together, our study demonstrated that HDEA could provide protection for skin cells against UV irradiation through the activation of the Nrf2 pathway. How it acts specifically at the molecular level remains to be discovered. Our study also provided an evidence-based idea that HDEA may be used as a novel therapeutic product for anti-oxidative stress and anti-photodamage that causes skin aging.

## 4. Materials and Methods

### 4.1. Materials

Dulbecco’s modified Eagle’s medium (DMEM), penicillin–streptomycin solution, and fetal bovine serum (FBS) were purchased from Gibco (Life technologies Korea, Seoul, Korea). 3-(4,5-dimethylthiazolyl-2)-2,5-diphenyltetrazoli-um bromide (MTT) and 2′,7′-di-chlorodihydrofluoresceindiacetate (H2DCF-DA) were purchased from Sigma–Aldrich (St. Louis, MO, USA). Anti-Nrf2, Anti-LaminB1, Anti-Procollagen1, Anti-MMP1, Anti-MMP3 and Anti-β actin antibody, Alexa Flour 488 goat anti-rabbit IgG and fluoroshield mounting medium with DAPI were purchased from Abcam (Cambridge, MA, USA). RNeasy Mini Kit was purchased from QIAGEN (Hilden, Germany). iScript™ Reverse Transcription Supermix and SsoFast™ EvaGreen^®^ Supermix were purchased from Bio-Rad (Hercules, CA, USA).

### 4.2. H. durvillei Extraction and Analysis

*H. durvillei* belongs to division Rhodophyta, class Fluoridephyceae, order Halymeniales, family *Halymenia* and species *Halymenia durvillei* [55]. *H. durvillei* seaweed was collected from Phetchaburi Coastal Fisheries Research and Development center in Amphor BanLam, Phetchaburi province, Thailand. For extraction, dried seaweeds were macerated in 95% ethanol for 7 days and evaporated at 40 °C to obtain ethanol extract (44.5 g) using rotary evaporation. After that, the ethanol fraction was partitioned with hexane, ethyl acetate, n-butyl alcohol and distilled water, respectively [27]. The ethyl acetate extract was obtained and dissolved in DMSO and diluted with the medium to obtain appropriate concentrations before being used for treating targeted cells.

### 4.3. Gas Chromatography-Mass Spectrometry (GC-MS) Analysis

To analyze active compounds of the ethyl acetate fraction of *H. durvillei*, the crude extract was investigated by GC-MS analysis at the Chemistry department of King Mongkut’s University of Technology Thonburi, Bangkok, Thailand [56]. Briefly, the ethyl acetate extract was analyzed by AGILENT GC-7890B/MSD-5977A system with a standard non-polar column HP-5 size of 30 mm × 0.25 mm ID × 0.25 µm film thickness. Helium was used as a carrier gas. The oven temperature started at 50 °C and increased to 250 °C using a heat rate of 5 °C/min. Compound identification was conducted by blasting with the National Institute of Standards and Technology (NIST) Mass Spectrometry Data Center.

### 4.4. Cell Culture

The human keratinocyte cell line HaCaT was purchased from Cell Lines Service (Heidelberg, Germany). The mouse fibroblast cell line L929 was purchased from ATCC. L929 and HaCaT cells were cultured in DMEM containing 10% FBS, 1% penicillin and streptomycin. Both cells were incubated at 37 °C with 5% CO_2_ in a humidified atmosphere [57].

### 4.5. UV Irradiation

UV irradiation was accomplished using a UV lamp comprising 280–320 nm of UVB and 320–360 nm of UVA (280–360 nm, G8T5E, 8 W, Sankyo Denki, Japan). UV intensity was measured by UVA & UVB radiometer RS232/USB and the irradiance intensity was 0.45 mW/cm^2^. L929 and HaCaT were seeded at the culture condition. To find an appropriate UV dose, UV irradiation was performed at 0, 10, 20, 40, 60 and 80 mJ/cm^2^. An appropriate UV dose was found to be 40 mJ/cm^2^ and used to irradiate both kinds of cells in all experiments.

### 4.6. Cell Cytotoxicity and Viability Assay

In order to test the cytotoxic effect of HDEA, L929 and HaCaT cells were seeded in 96 well plates at densities 1.5 × 10^4^ and 2.5 × 10^4^ cells/well, respectively, and cultured with HDEA for 24 h before incubating with 3-[4,5-dimethylthiazol-2-yl]-2,5-diphenyltetrazolium bromide (MTT) for 2 h. For cell viability assay, the cells were incubated with HDEA 24 h, washed with PBS, and then exposed to UV for 24 h. Then, the cells were incubated with MTT for 2 h. OD value was detected by a microplate reader at 570 nm [58].

### 4.7. Determination Intracellular Reactive Oxygen (ROS) Level

L929 and HaCaT cells were treated with different doses of HDEA and exposed to UV. The level of ROS was detected by the DCFH-DA assay kit. After UV radiation for 2 h, the cells were washed with PBS twice and incubated with H2DCF-DA for 45 min at 37 °C. Fluorescence intensity was immediately measured by a microplate reader TECAN spark at λ_exc_ 488 nm and λ_em_ 525 nm [59]. The fluorescence images were captured by Olympus IX83 Confocal Microscope.

### 4.8. Immunofluorescent Staining

For determining Nrf2 nuclear translocation, L929 and HaCaT cells were seeded onto a coverslip in 24-well plates. After reaching 70% confluence, the cells were treated with HDEA for 24 h. The cells were washed with PBS and fixed with 4% (*v*/*v*) formaldehyde for 15 min at RT and then washed with PBS. The cells were permeated with 0.5% Triton X-100 for 20 min, washed with PBS and blocked with 1% BSA for 30 min. The cells were then incubated in anti-Nrf2 antibody diluted in PBST with 1%BSA (1:200) at 4 °C, overnight and stained with Alexa Flour 488 goat anti-rabbit IgG in 1% BSA for 1 h, at RT in the dark. Finally, the cells were washed three times with PBS and mounted with fluoroshield mounting medium with DAPI [60]. The stained cells were imaged using Olympus’ BX53 upright microscope and analyzed using ImageJ.

### 4.9. Gene Expression Analysis by Quantitative RT-PCR

The mRNA levels encoding antioxidant enzymes including SOD1, CAT, GSTP1 and HmoxI were analyzed by quantitative RT-PCR. L929 and HaCaT cells were incubated with HDEA for 24 h before being exposed to UV for 6 h. Expression levels of the genes were quantified by qPCR after exposure to UV. Total RNA was extracted using the RNeasy Mini Kit according to the manufacturer’s instructions and measured by nanodrop. The cDNA was synthesized by using an iScript™ Reverse Transcription Supermix. Each qPCR was performed by using SsoFast™ EvaGreen^®^ Supermix, specific qPCR primers, and the converted cDNA. The qPCR primers are described in Table 2. The thermal conditions for PCR are as follows: initial denaturation at 95 °C for 3 min, followed by 44 cycles with denaturation at 56.1 °C and 66.7 °C in L929 and HaCaT, respectively, for 15 s. Annealing and elongation were performed at 60 °C for 60 s. The qPCR was performed in triplicate using independent RNA preparation. The data were quantified by the ΔΔCt method and represented as folds of the control sample [61].

### 4.10. Western Blot Analysis

For determining MMP1, MMP3 and procollagen1 expression levels, the cells were treated with HDEA and exposed to UV for 24 h. After UV irradiation, cellular proteins were extracted with RIPA buffer containing sodium fluoride and 1% PMSF, and the treated cells were detached using a cell scrapper. Cell lysates were sonicated for 2 min before centrifuging at 14,000 rpm at 4 °C for 10 min. For determining Nrf2 expression, the cells were harvested by Trypsin-EDTA solution. Nuclear proteins were extracted separately by employing the NEPERR Nuclear and Cytoplasmic Extraction Reagents according to the manufacturer’s instructions. The protein quantity in each cytoplasmic and nuclear fraction was estimated by the BCA assay kit. Equal amounts of proteins were electrophoresed on 12.5% SDS-PAGE, transferred to a nitrocellulose membrane and blocked for 2 h in Tris-buffered saline with 0.1% (*v*/*v*) Tween 20 (TBST) containing 5% bovine serum albumin (BSA). The membranes were incubated overnight at 4 °C with the primary antibody of Nrf2 at a 1:700 dilution, and LaminB1, MMP1, MMP3 and β-actin at a 1:1000 dilution in TBST. The membranes were washed with TBST and incubated with HRP-conjugated secondary antibody diluted at 1:5000 in TBST for 2 h at RT [62]. Protein bands were detected with Western blotting luminal reagent and visualized by Chemiluminescence detection. The relative amounts of proteins detected by each antibody were normalized with respective β-actin bands.

### 4.11. Statistical Analysis

GraphPad Prism 5 and Image J were used for data analyses. All data were analyzed by one-way ANOVA accompanied by Dunnett’s multiple comparison test for group comparison.

## 5. Conclusions

In the present study, the protective effect of HDEA against UV-induced photoaging was investigated in skin cells, L929 and HaCaT. The results indicated that HDEA could significantly reduce UV-induced ROS, MMP1 and MMP3 expressions in both L929 and HaCaT cells. In addition, HDEA also promoted procollagen restoration and Nrf2 nuclear translocation which led to the upregulations of its targeted antioxidant genes, including *GSTP1* and *HMOX1.* Thus, HDEA could be used as a pharmaceutical ingredient for counteracting photodamage due to UV irradiation and preventing skin aging. Further clinical trials are needed before they can be adopted for human usage.

## Figures and Tables

**Table 1:** Major compounds identified in HDEA by GC-MS (adapted with permission from Manohong et al., 2021 [27]).

	Compounds	RT (mins)	MW	Molecular Formula	Peak Area	% Peak Area
1	Hexadecanal	28.396	240.245	C_16_H_32_O	50617986	2.45
2	2-Undecanone	31.155	198.198	C_13_H_26_O	1.56 × 10^+8^	7.54
3	3-Cyclohexylpropionic Acid, 2,2,2 trifluoroethyl ester	32.615	238.118	C_11_H_17_F_3_O_2_	61606063	2.98
4	Palmitoleic acid	33.100	254.225	C_16_H_30_O_2_	77599531	3.75
5	(Z)-7-Hexadecene	33.237	224.25	C_16_H_32_	45720994	2.21
6	n-Hexadecanoic acid	33.637	256.24	C_16_H_32_O_2_	7.05 × 10^+8^	34.09
7	Shikimic acid	35.582	174.053	C_7_H_10_O_5_	45091910	2.18
8	1-Nonadecene	36.363	266.297	C_19_H_38_	4803209	2.32
9	2-butyl-5-hexyloctahydro-1H-Indene	36.938	264.282	C_19_H_36_	3.44 × 10^+8^	16.63
10	Octadecanoic acid	37.211	284.272	C_18_H_36_O_2_	95163245	4.6

RT: Retention time; MW: Molecular weight.

**Table 2:** Primer sequences used in the qPCR analyses.

Genes	Forward	Reverse
*CAT*	5′-CTTCGACCCAAGCAACATGC-3′	5′-GCGGTGAGTGTCAGGATAGG-3′
*SOD1*	5′-GATGACTTGGGCAAAGGTGG-3′	5′-TACACCACAAGCCAAACGACT-3′
*GSTP1*	5′-AAGTTCCAGGACGGAGACCT-3′	5′-ACATAGTCATCCTTGCCCGC-3′
*HMOX1*	5′-AGGGAATTCTCTTGGCTGGC-3′	5′-GACAGCTGCCACATTAGGGT-3′
*GADPH*	5′-GACAGTCAGCCGCATCTTCT-3′	5′-GCGCCCAATACGACCAAATC-3′
*Cat*	5′-TTGCCGTTCGATTCTCCACA-3′	5′-ATTTCACTGCAAACCCCCGA-3′
*Sod1*	5′-ATCCACTTCGAGCAGAAGGC-3′	5′-CTGATGGACGTGGAACCCAT-3′
*Gstp1*	5′-CGCGGCAAATATGTCACCCTC-3′	5′-CAGCAGGTCCAGCAAGTTGT-3′
*Hmox1*	5′-GGAAATCATCCCTTGCACGC-3′	5′-CTAGCAGGCCTCTGACGAAG-3′
*Gadph*	5′-CCCAGCTTAGGTTCATCAGGT-3′	5′-GGTCATGAGCCCTTCCACAA-3′

## Data Availability

The data supporting the conclusion in this study are available on request from the corresponding author.
